# Patterns, Predictors, and Outcomes of Falls Trajectories in Older Adults: The MOBILIZE Boston Study with 5 Years of Follow-Up

**DOI:** 10.1371/journal.pone.0106363

**Published:** 2014-09-03

**Authors:** Achille E. Tchalla, Alyssa B. Dufour, Thomas G. Travison, Daniel Habtemariam, Ikechukwu Iloputaife, Brad Manor, Lewis A. Lipsitz

**Affiliations:** 1 Harvard Medical School, Boston, Massachusetts, United States of America; 2 Division of Gerontology, Beth Israel Deaconess Medical Center, Boston, Massachusetts, United States of America; 3 Institute for Aging Research, Hebrew SeniorLife, Boston, Massachusetts, United States of America; 4 Department of Geriatric Medicine, University Hospital Center of Limoges, University of Limoges; EA 6310 HAVAE (Disability, Activity, Aging, Autonomy and Environment), Limoges, France; Cardiff University, United Kingdom

## Abstract

**Background:**

Falls may occur as unpredictable events or in patterns indicative of potentially modifiable risks and predictive of adverse outcomes. Knowing the patterns, risks, and outcomes of falls trajectories may help clinicians plan appropriate preventive measures. We hypothesized that clinically distinct trajectories of falls progression, baseline predictors and their coincident clinical outcomes could be identified.

**Methods:**

We studied 765 community-dwelling participants in the MOBILIZE Boston Study, who were aged 70 and older and followed prospectively for falls over 5 years. Baseline demographic and clinical data were collected by questionnaire and a comprehensive clinic examination. Falls, injuries, and hospitalizations were recorded prospectively on daily calendars. Group-Based Trajectory Modeling (GBTM) was used to identify trajectories.

**Results:**

We identified 4 distinct trajectories: No Falls (30.1%), Cluster Falls (46.1%), Increasing Falls (5.8%) and Chronic Recurring Falls (18.0%). Predictors of Cluster Falls were faster gait speed (OR 1.69 (95CI, 1.50–2.56)) and fall in the past year (OR 3.52 (95CI, 2.16–6.34)). Predictors of Increasing Falls were Diabetes Mellitus (OR 4.3 (95CI, 1.4–13.3)) and Cognitive Impairment (OR 2.82 (95CI, 1.34–5.82)). Predictors of Chronic Recurring Falls were multi-morbidity (OR 2.24 (95CI, 1.60–3.16)) and fall in the past year (OR 3.82 (95CI, 2.34–6.23)). Symptoms of depression were predictive of all falls trajectories. In the Chronic Recurring Falls trajectory group the incidence rate of Hospital visits was 121 (95% CI 63–169) per 1,000 person-years; Injurious falls 172 (95% CI 111–237) per 1,000 person-years and Fractures 41 (95% CI 9–78) per 1,000 person-years.

**Conclusions:**

Falls may occur in clusters over discrete intervals in time, or as chronically increasing or recurring events that have a relatively greater risk of adverse outcomes. Patients with multiple falls, multimorbidity, and depressive symptoms should be targeted for preventive measures.

## Introduction

Falls are common among older persons [1], [2] and rank among the 10 leading causes of death in the United States, resulting in more than $19 billion in health care costs annually [3]. Falls account for approximately 10% of visits to an emergency department and 6% of hospitalizations among Medicare beneficiaries [4].

Although scientific evidence supports associations between a number of risk factors and falls [5], efforts to translate these findings into effective fall prevention strategies have been limited [6]. A Cochrane review showed that multifactorial interventions significantly reduce the rate of falls in varying degrees [7], [8] but it may be difficult to identify the appropriate group to target for interventions. It is possible that some people may experience falls as random, unpredictable events, while others may have patterns of falls that are indicative of potentially modifiable risks factors and are predictive of adverse outcomes. Knowing the patterns, risks, and outcomes of falls trajectories may help clinicians plan appropriate preventive measures. To our knowledge there are no longitudinal studies that have observed long-term trajectories of falls and determined their predictors and outcomes.

We hypothesized that: 1- there are distinct clinical patterns of falls trajectories ranging from no falls, random falls, clusters of falls, progressively increasing numbers of falls, and chronic recurring falls; 2- baseline “predictive factors”, such as chronic illnesses associated with these patterns could be identified; and 3- clinically important health outcomes would be worse in those with chronically elevated fall rates. Therefore, we examined a unique longitudinal database from the MOBILIZE Boston Study (which stands for The Maintenance Of Balance, Independent Living, Intellect, and Zest in Elderly (MBS)), which rigorously collected falls calendar data from a cohort of community-dwelling elderly people over a 5 year period. We used this database to identify subgroups of people with distinct falls trajectories, identify baseline characteristics associated with these trajectories, and determine their coincident clinical outcomes.

## Materials and Methods

### Ethics Statement

The MOBILIZE Boston Study Trajectory (MBSTraj), Protocol Number: 13-029 was reviewed and approved by the Hebrew SeniorLife Institutional Review Board (IRB) at the Hebrew Rehabilitation Center (HRC) in Boston. Written informed consent was obtained from each participant at each phase of MOBILIZE Boston Study. The study was conducted according to the principles of Helsinki Declaration.

### Study participants

Study participants were women and men aged 70 years and older living in the community in Boston and nearby suburbs. Recruitment and enrollment took place from September 2005 to January 2008 within a defined geographic area bounded by a 5-mile radius around the Institute for Aging Research at HRC in Boston. The sampling area was chosen to capture a diverse urban and suburban population, to increase likelihood of recognition of the study center, and to minimize transportation burden. Details of the study methods were published previously [9], [10]. Initial eligibility was based on age 70 years or older, ability to walk 20 feet without personal assistance, ability to communicate in English, and the expectation of staying in the area for 2 years. Following the initial recruitment visit, study staff contacted prospective enrollees by telephone to confirm eligibility and schedule the baseline home and clinic visits. During the home visit, written informed consent was obtained and participants were screened and excluded for moderate or severe cognitive impairment using the Mini-Mental State Examination (MMSE score, <18) [11], [12].

### Falls and Clinical Outcomes Assessments

During the Follow-up, a fall was defined as unintentionally coming to rest on the ground or other lower level not as a result of a major intrinsic event (e.g., myocardial infarction, stroke, or seizure) or an overwhelming external hazard (e.g., hit by a vehicle) [13]. Participants were instructed to complete and return monthly falls calendar postcards designed to be posted on a refrigerator. On the postcards, participants were to record an *F* for each fall on the day it occurred and an *N* on days when No Falls occurred. This approach has been well-validated for use in epidemiological cohort studies [14]. The calendar postcards also included questions about whether the participant experienced a hospitalization during the preceding month. Research staff monitored the return of the calendars and on any given month, approximately one-third of participants were called for missing or incomplete calendars. All subjects who reported falls were also called to determine the circumstances of the fall and clinical outcomes whether any injuries (e.g. fractures) and hospital visits were incurred.

### Covariates

Covariates included sociodemographic characteristics, physiologic risk factors, health status, and amount of physical activity. Sociodemographic characteristics assessed in the home interview included age, sex, race (self-identified), and years of education. Cognitive status was assessed using the MMSE, scored 0–30 [12]. We used the validated Physical Activity Scale for the Elderly (PASE) to measure physical activity in the previous week [15]. Participants were asked about physician-diagnosed major medical conditions. Details of the study methods were published previously [9], [10]. During the clinic visit, several measures were calculated. Diabetes was defined using an algorithm based on self-reported diabetes, use of antidiabetic medications, and laboratory measures from the baseline clinic visit including random glucose (200 mg/dL) and hemoglobin A_1c_ (7%). Depression was assessed using the Eaton method based on a modification of the 20-item Centers for Epidemiologic Studies Depression scale [16]. Body mass index (calculated as weight in kilograms divided by height in meters squared) was calculated from measured height and weight. Standing balance was scored using 4 timed tests (side-by-side, semi-tandem, tandem, and 1-leg stands) [17]. For the timed chair stands test, participants were asked to fold their arms across their chest and stand up and down from a chair 5 times as quickly as possible [17]. Gait speed was based on the shortest time of 2 trials of a usual-paced 4-meter walk [17].

### Data Analysis

**Figure 1:**
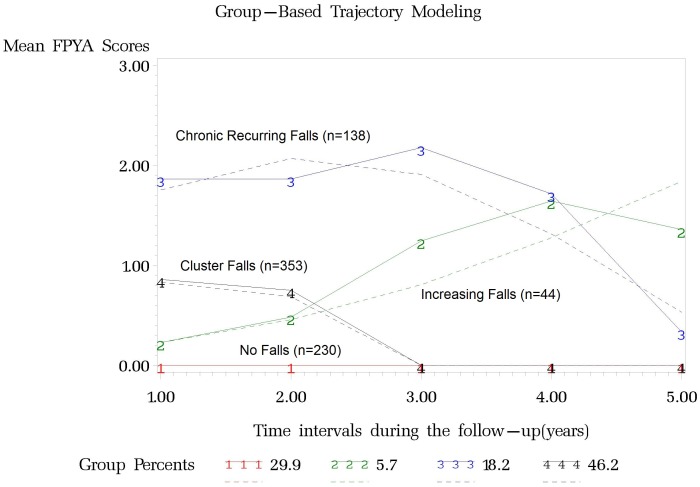
Patterns of Falls Trajectories over 5 years in Older Adults (The MOBILIZE Boston Study). —Solid lines are estimated trajectories. ---- Dashed lines are observed trajectories for each group.

To identify clinically distinct trajectories of falls, we use Group-Based Trajectory Modeling (GBTM) [18]. This method allowed us to simultaneously estimate probabilities for multiple trajectories rather than a single mean for the population, as is the case for traditional regression or growth-curve models. We used SAS software, and the PROC TRAJ macro (http://www.andrew.cmu.edu/user/bjones) [19], [20], a closed-source module developed specifically for use with SAS software, which fits a semiparametric mixture model to longitudinal data with the use of the maximum-likelihood method. It is possible that some people may experience falls as random, unpredictable events, while others may have patterns of falls that are indicative of potentially modifiable risks factors and are predictive of adverse outcomes. Knowing the patterns, risks, and outcomes of falls trajectories may help clinicians plan appropriate preventive measures. The metric for defining the trajectory was years into the study. The number of falls that each participant reported each month was summated for each year over the 5 years study period and quantified as a “Falls Per Year of Aging” Score (FPYA Score). For each one year time interval, No Falls equaled FPYA 0, one fall equaled FPYA 1, two falls equaled FPYA 2 (recurrent falls), and three or more falls equaled FPYA 3 (high number of recurrent falls). FPYA scores were examined as a function of different time periods from 1 to 5 years long, independent of the chronological time at which these periods started (e.g., a 3 year period could start at year 1 and include years 1–3, year 2 and include years 2–4, or year 3 and include years 3–5 of the study). PROC TRAJ was used with the follow-up time metric from 1 to 5 years long independent of their age. So interval time was 1-year and Falls Per Year Aging (FPYA) was scored with 1-year follow-up time metric. FPYA (Falls per year aging) as the y-axis in Figure 1 represents the average of fall rate per year as a function of different intervals in time (shown on the x-axis). These scores were modeled as a censored normal distribution. We used the Bayesian Information Criterion (BIC) to test from three to six trajectories and to determine whether each trajectory was best fit by intercept only (i.e., constant) or by linear, quadratic, or cubic terms [21]. The final model was selected based on a combination of the Bayesian information criterion (BIC; where the value closest to 0 indicates the best-fitting model) and by estimated trajectory group proportions that were sufficiently large (e.g., 0.05) [22]. These analyses were repeated after adjustment for age, sex, race or ethnic group, years of education, chronic conditions such as hypertension, stroke, diabetes mellitus, hyperlipidemia, cognitive status and previous falls at baseline using the Proc Traj software “risk” command. The proportions of older adults classified according to each trajectory, the mean probability of assignment, and the proportions with poor fit were based on the original data, and 95% confidence intervals were estimated with the use of 1000 bootstrap samples [18]. Missing data on the trajectory modeling and on time-varying variables were handled with a maximum likelihood approach [18] together with the missing-at-random assumption, which assumes that for each individual, the likely values for missing data on the trajectory and time-varying variables can be estimated from other available observed data. This approach uses available information on each case for constructing the trajectories rather than deleting individuals with missing observations.

After determining the most appropriate GBTM, group status for each individual was obtained to identify relevant predictors of each falls trajectory group. An individual was assigned to the trajectory group in which he or she was most likely to be, as determined by the group posterior probabilities from the final model. After using the “risk” command in Proc Traj software to identify predictors with beta coefficients and p values, we confirmed the findings and computed odds ratios using multinomial analyses. The outcome variable was falls trajectory group membership, and the testable predictors were sociodemographic characteristics, physiologic risk factors, health status, and amount of physical activity. The models were estimated using PROC LOGISTIC in SAS version 9.3. Partial *R* ^2^ was calculated for statistically significant predictors using *R* ^2^max values [23], [24] and the Area Under the Curve was calculated for the model discrimination [25]. We assessed relevant clinical outcomes such as injurious falls, falls resulting in fractures, and hospital visits according to falls trajectories. All analyses were performed using SAS software, version 9.3 (SAS Institute Inc, Cary, North Carolina). A two-sided P value of less than 0.05 was considered indicative of statistical significance.

## Results

### Participants

Eight hundred nine individuals met study inclusion. Forty four of them older adults were missing data and therefore excluded from the analysis. **Table 1** summarizes the demographic and health characteristics of the study sample at baseline. Of the 765 older adults included in this study, 276 (36.1%) were male and 489 (63.9%) female, 156 (20.4%) were nonwhite, 245 (32.0%) met the PASE score of low intensity physical activity. On average, the cohort was 78.1±5.4 of age (range 64–97). Over five years, 90 (11.8%) older adults died during the follow-up period, with an incidence rate of 24 (95% CI 13–34) per 1,000 person-years.

**Table 1:** Summary of Baseline Characteristics (The MOBILIZE Boston Study).

Demographic Characteristics and Risk Factors	Total sample n = 765^a^	
	No.	%
Age, mean (SD), y	78.1	(5.4)
Women,	488	63.8
Race,		
White	609	79.6
Nonwhite	156	20.4
Years of Education, mean (SD), y	14.8	(6.1)
Body mass index, kg/m^2^, b		
<25	233	30.4
25–29.9	297	38.8
≥30	206	26.9
Fell in past year,	289	37.8
Comorbidities ≥2,	491	64.2
Hypertension,	607	79.3
Previous stroke,	72	9.4
Diabetes mellitus,	172	22.5
Hyperlipidemia,	469	61.3
Congestive heart failure,	38	5.0
Mini-Mental State Examination score <24, c	154	20.1
CES-D Revised score, mean (SD), points	11.0	(11.2)
Physical activity score, d		
0–66	245	32.0
66.01–124	262	34.3
124.01–559	258	33.7
Impaired balance, score <4 out 7, e	318	41.6
Slow gait speed, <0.78 m/s, f	189	24.7
Slow chair stands, >16.37 s, g	95	12.4

a Forty four of the 809 older adults were missing data and therefore excluded from the analysis.

b Body mass index is calculated as weight in kilograms divided by height in meters squared.

c Mini-Mental State Examination (MMSE) cut off point for cognitive impairment [11], [12].

d Physical activity tertiles measured using the Physical Activity Scale for the Elderly [15].

e Balance score was based on 4 progressively difficult stands: feet side by side, semi-tandem, tandem, and 1-leg stand [17].

f Slow gait speed (m/s) is the lowest 25% based on time of lowest of 2 usual-paced 4-meter walks [17].

g Highest quartile (slowest performance) of time to complete 5 repeated chair stands [17].

### Determining Falls Trajectories over 5 years

The BIC values and estimated proportions for the 3-, 4-, and 5-group GBTM models shown in **Table 2** were used to determine the best fit. A 6-group model was tested but failed to converge. Consequently, the 4 group model was selected for each fall trajectory group. Additional diagnostic criteria for judging the adequacy of a GBTM demonstrated that the 4 group model performed well based on the Nagin criteria [18]. **Figure 1** illustrates each of the 4 trajectories along with the average raw group data at each point. The 4 distinct trajectories were: Cluster Falls (46.1%; 95CI, 40.0%–48.8%; n = 353) *P*<0.001, Increasing Falls (5.8%; 95CI, 3.7%–11.4%; n = 44) *P*<0.001, Chronic Recurring Falls (18.0%; 95CI, 12.7%–19.1%; n = 138) *P*<0.001, and No Falls (30.1%; 95CI, 28.8%–35.6%; n = 230) *P*<0.001. **Table 3** presents descriptive data for each falls trajectory group.

**Table 2:** Bayesian Information Criterion (BIC) Values and Predicted Group Proportions for Group-Based Trajectory Models.

Model	No. of Groups	BIC	Predicted Group Proportions				
			Group 1	Group 2	Group 3	Group 4	Group 5
1	3	−2385.99	0.272	0.495	0.233		
2	4	−2350.01	0.302	0.465	0.058	0.175	
3	5	−2350.87	0.318	0.452	0.044	0.074	0.112

**Table 3:** Summary of Baseline Characteristics by Falls Trajectory Profiles Derived From Group-Based Trajectory Modeling (The MOBILIZE Boston Study, No. = 765^a^).

Characteristics and Risk Factors	Trajectory Group^b^								*P* Value^c^
	No Falls n = 230, 30.1%		Cluster Falls n = 353, 46.1%		Increasing Falls n = 44, 5.8%		Chronic Recurring Falls n = 138, 18.0%		
	No.	%	No.	%	No.	%	No.	%	
Age, mean (SD), y	78.1	(5.4)	77.9	(5.4)	76.5	(4.7)	78.8	(5.6)	0,71
Women,	142	61.6	232	65.7	29	66.7	85	61.5	0,73
Race,									
White	155	67.2	282	79.9	35	79.5	137	99.1	<0.001
Nonwhite	75	32.8	71	20.1	9	20.5	1	0.9	
Years of Education	14.3	(8.3)	15.0	(5.2)	14.3	(2.4)	15.8	(2.3)	0.27
Body mass index, kg/m^2^, d									
<25	71	31.0	108	30.6	3	7.7	51	37.3	
25–29.9	97	42.1	116	40.8	22	50.0	62	45.2	<0.05
≥30	62	26.9	101	28.6	19	42.3	24	17.5	
Fell in past year,	28	11.9	153	43.3	6	13.7	102	73.9	<0.001
Comorbidities ≥2,	121	52.6	231	65.5	32	73.7	107	77.5	<0.01
Hypertension,	183	79.6	276	78.2	37	83.3	111	80.4	0.08
Stroke,	23	10.0	31	8.8	0	0.0	18	12.8	0.24
Diabetes mellitus,	54	23.3	75	21.3	30	68.4	13	9.4	<0.001
Hyperlipidemia,	136	59.2	223	63.3	37	83.3	73	52.9	<0.01
Congestive heart failure,	10	4.4	20	5.7	0	0.0	8	5.5	0.6
Mini-Mental State Examination score <24, e	36	15.7	44	12.5	7	16.7	67	4.9	<0.05
CES-D Revised score, mean (SD), points	6.2	(6.8)	11.6	(11.6)	16.3	(14.8)	14.7	(11.7)	<0.001
Physical activity score, f									
0–66	67	29.1	122	34.6	24	53.9	32	23.1	
66.01–124	70	30.4	129	36.5	15	34.6	48	35.0	<0.01
124.01–559	93	40.5	102	28.9	5	11.5	58	41.9	
Impaired balance, score <4 out 7, g	86	37.6	158	44.7	8	19.1	66	47.6	<0.05
Slow gait speed, <0.78 m/s, h	73	31.7	72	20.5	7	15.9	37	27.1	<0.01
Slow chair stands, >16.37 s, i	23	10.1	55	15.6	4	9.5	13	9.4	0.11

a Forty four of the 809 older adults were missing data and therefore excluded from the analysis;

b Estimated from Group-Based Trajectory Modeling (GBTM) [18].

c Global test: χ2 or Fisher's exact test for binary variables; analysis of variance for continuous variables.

d Body mass index is calculated as weight in kilograms divided by height in meters squared.

e Mini-Mental State Examination (MMSE) cut off point for cognitive impairment [11], [12].

f Physical activity tertiles measured using the Physical Activity Scale for the Elderly [15].

g Balance score was based on 4 progressively difficult stands: feet side by side, semi-tandem, tandem, and 1-leg stand [17].

h Slow gait speed (m/s) is the lowest 25% based on time of lowest of 2 usual-paced 4-meter walks [17].

i Highest quartile (slowest performance) of time to complete 5 repeated chair stands [17].

### Coincident Clinical Outcomes of Falls Trajectories

**Figure 2:**
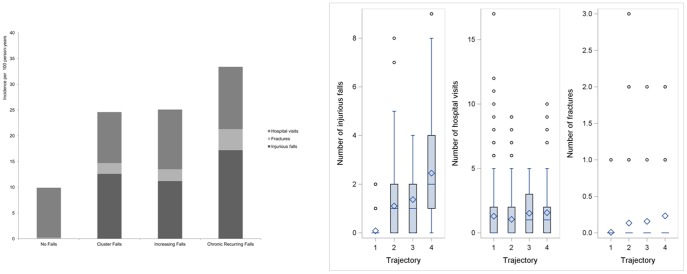
Clinical Outcomes according to Falls Trajectory group membership (The MOBILIZE Boston Study). **A.** Incidence Rate of Clinical Outcomes according to Falls Trajectory Group. **B.** Box plot showing Averages of Clinical Outcomes according to Falls Trajectory Group: *Trajectories*: 1 = No Falls; 2 = Cluster Falls; 3 = Increasing Falls; 4 =  Chronic Recurring Falls. *Injurious Falls*: 2&4 p<0.001 (S); 3&4 p<0.001 (S); All others NS. *Hospital visits*: All are NS. *Fractures*: 1&2 p<0.001 (S), 1&3 p<0.001 (S), 1&4<0.0001, All others are NS.

**Figure 2** panel A shows the distribution of the incidence of clinical outcomes namely injurious falls, fractures and hospital visits for each falls trajectory and summarized below. The average of clinical outcomes and incidence followed the same distribution in falls trajectories. The incidence of *Injurious Falls* was 172 (95% CI 111–237) per 1,000 person-years in Chronic Recurring Falls trajectory group, 126 (95% CI 98–169) per 1,000 person-years in Cluster Falls trajectory group, and 112 (95% CI 51–267) per 1,000 person-years in Increasing Falls trajectory group. The incidence of *Fractures* was 41 (95% CI 9–78) per 1,000 person-years in the Chronic Recurring Falls trajectory group, 21 (95% CI 7–38) per 1,000 person-years in the Cluster Falls trajectory group, 23 (95% CI 10–67) per 1,000 person-years in Increasing Falls trajectory group, and 2 per 1,000 person-years in No Falls trajectory group. The incidence of *Hospital visits* was 121 (95% CI 63–169) per 1,000 person-years in Chronic Recurring Falls trajectory group, 116 (95% CI 20–207) per 1,000 person-years in Increasing Falls trajectory group, 99 (95% CI 68–130) per 1,000 person-years in Cluster Falls trajectory group and 97 (95% CI 58–134) per 1,000 person-years in the No Falls trajectory group. **Figure 2** panel B shows that during the 5-year follow-up, the mean number of injurious falls was significantly higher in the “Chronic Recurring Falls” trajectory group. The mean number of fractures was also higher in the “Increasing Falls” and “Chronic Recurring Falls” trajectory groups compare to the “No falls Group”.

### Predictors of Falls Trajectories

Multinomial logistic regression revealed that specific baseline characteristics predicted membership within each of the three “faller” trajectory groups as compared to the no falls trajectory group are shown in **Table 4**. Predictors of the Cluster Falls trajectory were faster gait speed and falls in past year. Predictors of the Increasing Falls trajectory were Diabetes Mellitus (DM) and Cognitive Impairment. Predictors of Chronic Recurring Falls were two or more comorbid chronic conditions and falls in the past year. Symptoms of depression were predictive of all groups.

**Table 4:** Adjusted Odds Ratios for every unit increase or Category Change in the Predictors of Falls Trajectory: Results from Multinomial Logistic Regressions at Baseline (n = 765^a^), The MOBILIZE Boston Study.

Predictor	Falls Trajectory Group^b^								
	Cluster Falls^1^ (n = 353, 46.1%)			Increasing Falls^2^ (n = 44, 5.8%)			Chronic Recurring Falls^3^ (n = 138, 18.1%)		
	OR^c^	95CI	*P* Value^d^	OR^c^	95CI	*P* Value^d^	OR^c^	95CI	*P* Value^d^
Age, y	0.99	0.96, 1.04	0.94	1.01	0.88, 1.05	0.80	1.04	0.97, 1.12	0.21
Sex									
Male	1.00	Referent		1.00	Referent		1.00	Referent	
Women	1.31	0.88, 1.96	0.19	2.97	0.42, 2.87	0.10	1.85	0.92, 3.74	0.09
Race									
Nonwhite	1.00	Referent		1.00	Referent		1.00	Referent	
White Race	1.16	0.94, 1.43	0.17	1.07	0.67, 1.69	0.21	1.64	0.98, 2.67	0.06
Years of Education	1.02	0.99, 1.04	0.29	0.98	0.91, 1.05	0.49	1.04	0.99, 1.09	0.11
Body mass index, kg/m^e^	1.05	0.80, 1.37	0.73	1.04	0.56, 1.95	0.97	1.02	0.62, 1.68	0.94
Fell in past year	3.52	2.16, 6.34	<0.001	1.69	0.53, 5.38	0.16	3.82	2.34, 6.23	<0.001
Comorbidities ≥2	2.06	0.95, 3.14	0.08	2.52	0.89,7.20	0.40	2.24	1.34, 5.32	0.015
Hypertension	0.70	0.55, 0.91	0.16	1.25	0.62, 2.53	0.80	0.85	0.40, 1.75	0.11
Stroke	0.83	0.46, 1.48	0.35	-	-	-	2.08	0.67, 6.45	0.20
Diabetes mellitus	0.69	0.40, 1.18	0.18	4.30	1.40, 13.3	<0.001	0.20	0.03, 1.27	0.08
Hyperlipidemia	1.19	0.84, 1.68	0.13	2.60	0.84, 8.06	0.32	2.05	0.98, 4.30	0.06
Congestive heart failure	1.24	0.56, 2.72	0.93	-	-	-	0.76	0.15, 3.91	0.75
Cognitive status^f^									
MMSE≥24	1.00	Referent		1.00	Referent		1.00	Referent	
MMSE<24	0.83	0.44, 1.58	0.57	2.82	1.34, 5.82	0.039	0.44	0.11, 1.66	0.21
CES-D Revised score^g^	1.03	1.01, 1.05	0.006	1.04	1.00, 1.07	0.013	1.07	1.03, 1.10	<0.001
Physical activity score^h^									
≥66	1.00	Referent		1.00	Referent		1.00	Referent	
<66	1.02	0.66, 1.37	0.92	1.23	0.70, 2.17	0.57	1.82	0.86, 3.87	0.12
Impaired balance, score <4 out 7^i^	1.29	0.84, 1.97	0.24	0.74	0.27, 2.00	0.26	1.94	0.91, 4.15	0.09
Fast gait speed, >0.78 m/s^j^	1.69	1.50, 2.56	0.012	0.84	0.21, 2.08	0.47	1.61	0.66, 3.85	0.29
Slow chair stands, >16.37 s^k^	1.18	0.65, 2.18	0.58	1.12	0.25, 5.26	0.75	1.04	0.52, 2.08	0.75

Abbreviations: 1, Model 1; 2, Model2; 3, Model3; OR, Odds Ratio; 95%CI, 95% Confidence Interval;

a Forty four of the 809 older adults were missing data and therefore excluded from the analysis;

b Estimated from Group-Based Trajectory Modeling (GBTM) [18].

c No Falls group was the reference category.

d Two-sided *P* value.

e Body mass index is calculated as weight in kilograms divided by height in meters squared;

f MMSE (Mini-Mental State Examination Scale) <24 [11], [12], Cognitive impairment;

g CES-D, Center for Epidemiological Studies Depression Scale [16];

h Low physical activity, <66.01 PASE Score (using the Physical Activity Scale for the Elderly) [15];

i Balance score was based on 4 progressively difficult stands: feet side by side, semi-tandem, tandem, and 1-leg stand [17];

j Fast gait speed (m/s) is the highest 25% based on time of fastest of 2 usual-paced 4-meter walks [17];

k Highest quartile (slowest performance) of time to complete 5 repeated chair stands [17]. R^2^max [23], [24] (Model1: 0.32; Model2: 0.36; Model3: 0.57) AUC [25] (Model1: 0.80; Model2: 0.89; Model3: 0.92).

## Discussion

In this 5-year prospective cohort study, we examined the course of falls in community-dwelling older adults. We found four clinically distinct trajectories of falls that we have labeled: cluster falls, increasing falls, chronic recurring falls, and no falls. We found that Diabetes mellitus, cognitive impairment, fast gait speed, falls in the past year at baseline, and multi-morbidity are predictors of these trajectories. Importantly, these trajectories were associated with adverse outcomes including injurious falls and fractures. Notably, people with chronic recurrent falls had the highest rate of injurious falls and fractures. There was a marginally significant increase in hospitalizations among those with increasing falls.

The number of falls that each participant reported each month was summated for each year over the 5 years study period and quantified as a “Falls Per Year of Aging” Score (FPYA Score). For each one year time interval, No Falls equaled FPYA 0, one fall equaled FPYA 1, two falls equaled FPYA 2 (recurrent falls), and three or more falls equaled FPYA 3 (high number of recurrent falls). These scores were distributed according the censored normal distribution and their modeling identified 4-groups.

The major utility of the 4-group solution is in the ability of these 4 groups to predict adverse outcomes. In addition, the 4-group trajectory solution identified some risk factors to target for interventions. These patterns of falls had a few distinguishing risk factors described above. Some of these are potentially modifiable, such as diabetes for increasing falls and comorbid conditions for Chronic recurring falls. The greater proportion of fast walkers in the cluster group may represent people who are less careful or have more acute condition rather than chronic diseases (e.g. these comorbity scores are lower than other fall groups). We believe that falls incidence declines in the chronic recurrent falls group beginning in year 4 because people who had recurrent falls had clinically adverse events like hospital visits or fractures which reduced their mobility and chance of falling. Recurrent falls over 3-years may signal the beginning of functional decline.

This study is unique in demonstrating that not all falls have the same clinical implications. As we have shown, some may occur in relatively short-lived clusters, perhaps from an acute illness or exposure to a new drug or environmental hazard; some may increase in frequency over time, possibly due to the progression of a disease or disability; and some may recur at a relatively high rate over time. Each of these presentations appears to have a distinct set of risk factors and in some circumstances different prognostic implications. For example, chronic recurrent falls seem to occur in people with multiple co-morbidities (multi-morbidity) [26], [27] and depressive symptoms, and, as might be expected, are associated with a relatively high rate of injuries and fractures.

Although a high incidence of falls has been reported in older populations [1], little is known about the progression of falls over a long period of time. To our knowledge there are no longitudinal studies that observed the trajectories of fallers. While some falls are isolated, non-recurring events, others may mark the beginning of a progressive down-hill course in which the injuries and fear associated with an initial fall may precipitate recurrences and spiraling functional decline. A previous study showed that illnesses and injuries leading to either hospitalization or restricted activity are strongly associated with the initial onset of disability [28]. Another study extended this earlier work by demonstrating that exposure to intervening illnesses and injuries are also associated with the subsequent course of disability [29]. Therefore, it is important to distinguish those falls that are likely to be isolated episodes from those that lead to further disability. A Cochrane review highlighted the difficulty identifying an appropriate group for preventive efforts as a result of this heterogeneity in the presentation and subsequent course of falls in older populations [7], [8].

Our study has several limitations. 1) We used baseline clinical characteristics that were obtained up to 5 years before the falls occurred, so we were unable to identify risk factors at the time the falls. This limited our ability to determine whether cluster falls were due to acute illnesses, drugs, or environmental hazards, and whether other patterns followed the course of chronic diseases. 2) Some of our participants experienced falls before the study began. Thus, these falls were left censored in our analysis. It is therefore possible that some of our non-fallers experienced fall episodes before the study began. We performed secondary analyses, stratifying previous fallers by non-fallers and found similar patterns. Unfortunately, the sample sizes of these subgroups were too small to draw meaningful conclusions. 3) Finally, because our study participants were only from a Massachusetts geographic area within a 5-mile radius of the Institute for Aging Research, the cohort may not be generalizable. However, a comparison of the demographics of MBS study participants to the US Census showed comparable distributions by sex and racial group in the population aged 70 and older [9].

In summary, our study provides evidence suggesting four clinically distinct patterns of falls over five years among community-dwelling older adults. Each of these patterns has a distinct set of independent risk factors. Compared to all other groups, chronic recurring falls are associated with the highest rate of injuries. Older adults with recurrent falls should be targeted by health care professionals to identify their underlying cause and implement interventions to prevent subsequent injury.
